# Supplementary material for: Characterization of a Topically Testable Model of Burn Injury on Human Skin Explants
Source: Int J Mol Sci. 2020 Sep 22;21(18):6956. doi: 10.3390/ijms21186956 (PMC7554828; doi:10.3390/ijms21186956)

Supplementary Materials

Figure S1. Kinetics of a-SMA, Collagen I, and Collagen III dermal expression in *ex vivo* cultured human skin explants submitted to 10s-long experimental injury. Immunostainings of the dermal proteins a-SMA (a), Collagen I (b), and Collagen III (c) were performed on human skin explants harvested at different time points post-burn (d5, d10 and d14). Results were compared to those obtained in non-lesioned skin explants (d0). No significant difference was observed when comparing non-lesioned vs lesioned skin explants, irrespective of the time point considered following burn injury. Results obtained for each condition are representative of 3 series of experiments performed on skin explants derived from 3 donors. Scale bar: 100 µm.

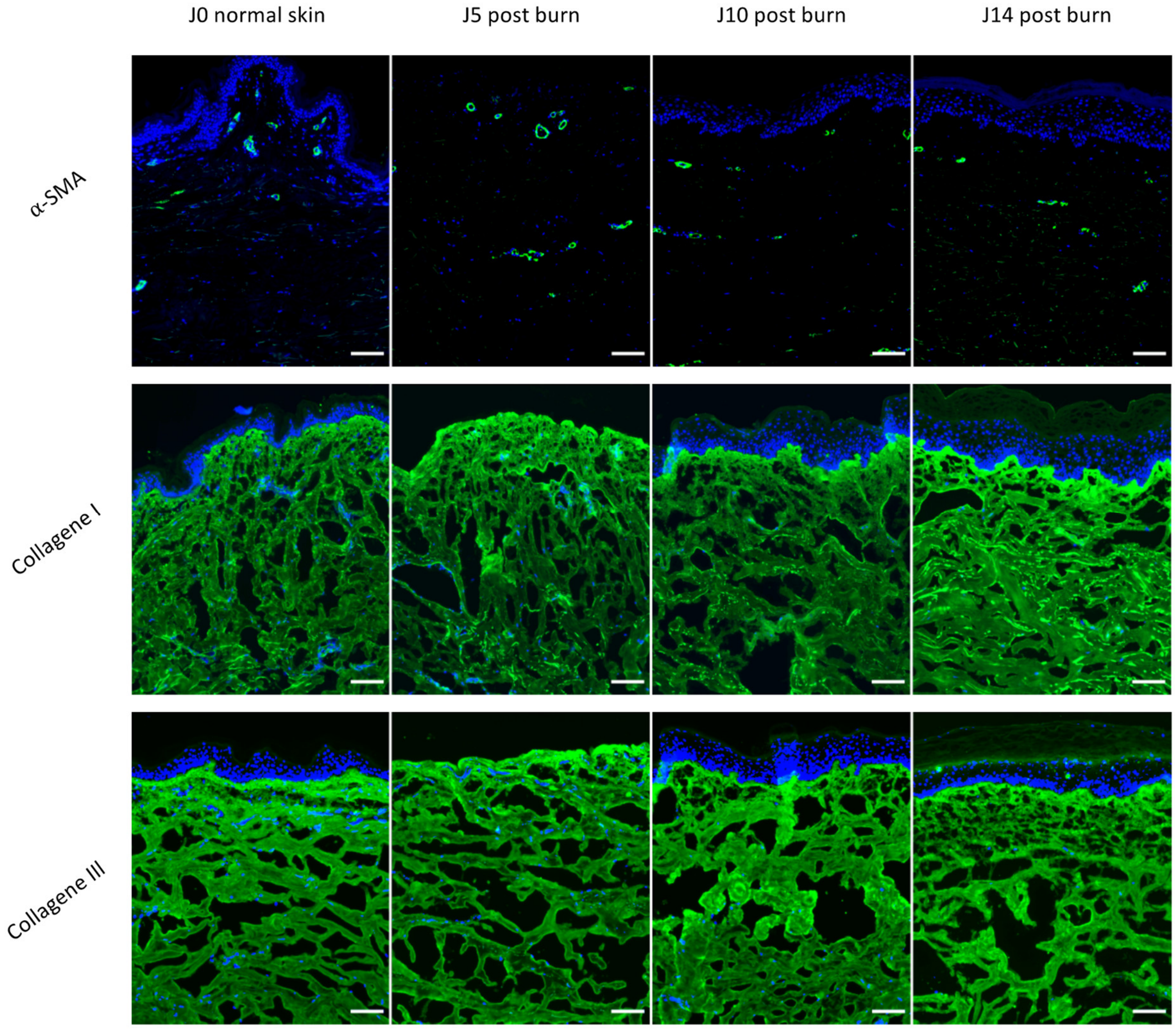

Figure S2. Increase of HLA class II staining at the edge of the lesion at d5 post-burn in *ex vivo* cultured human skin explants submitted to 10s-long experimental injury. Immunostainings of HLA class II molecules were performed on human skin explants harvested at different time points post-burn (d5, d10 and d14) (a). A local increase of HLA class II immunostaining was observed at d5 post-burn in the dermis and epidermis at the edges of the injured area. Such an increase is transient as immunostaining intensity returns to baseline levels at d8 onwards. Black dashed lines delineate the burnt area. (b) High magnification views of HLA class II immunostainings observed at the edges of the burnt area at d5 post-burn. Results obtained for each condition are representative of 3 series of experiments performed on skin explants derived from 3 donors. Scale bar: 100 μm.

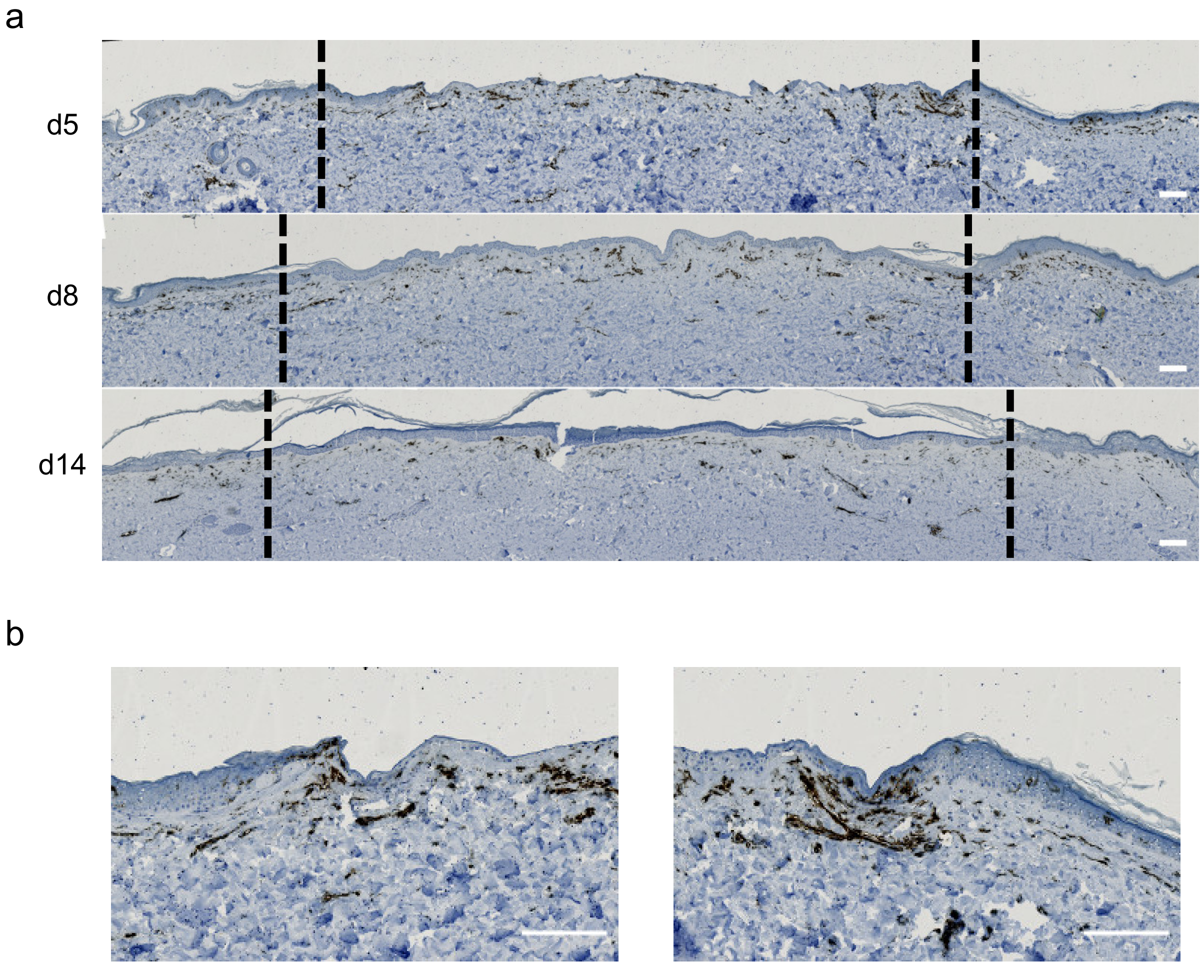

Supplement: Supplementary file 1 [file ijms-21-06956-s001.pdf]
